# Preparing for Dengue Vaccine Introduction: Recommendations from the 1st Dengue v2V International Meeting

**DOI:** 10.1371/journal.pntd.0002261

**Published:** 2013-09-26

**Authors:** Joseph Torresi, Roberto Tapia-Conyer, Harold Margolis

**Affiliations:** 1 Austin Health and University of Melbourne, Victoria, Australia; 2 Carlos Slim Health Institute, Mexico City DF, Mexico; 3 Centers for Disease Control and Prevention, San Juan, Puerto Rico; University of Rhode Island, United States of America

## Introduction

**Figure 1:**
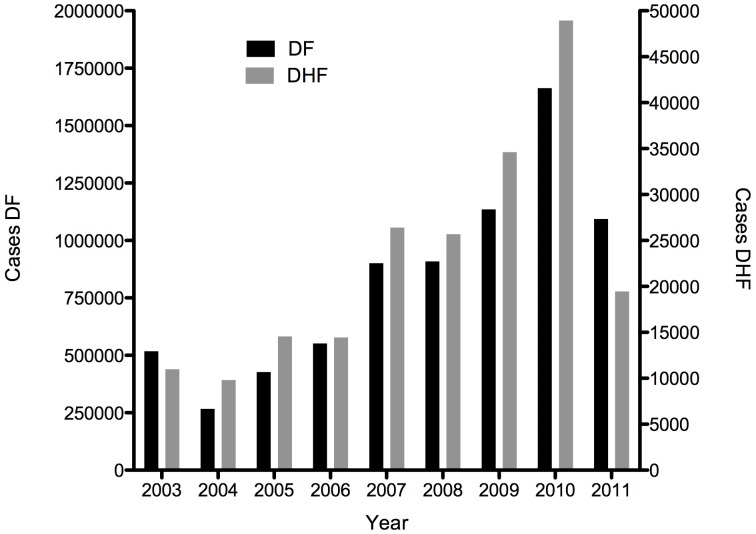
Increasing number of cases of dengue and dengue haemorrhagic fever (DHF) in Latin America, 2003–2011. This graph was produced by the authors using data available from PAHO [25].

Dengue is a major public health concern, resulting in significant morbidity, mortality, and economic and human costs, particularly in developing countries [1]–[4]. Approximately 40% of the world's population lives in areas where dengue virus (DENV) transmission occurs, and dengue has recently become a threat in previously unaffected regions [5]. Cases of dengue, and its severe form, dengue haemorrhagic fever (DHF), are increasing in endemic areas. For example, in Latin America, cases of both dengue and DHF have steadily increased since 2003 (Figure 1). Integrated vector control, although used widely, has been largely ineffective in preventing or interrupting endemic DENV transmission and also raises concerns regarding insecticide resistance, toxicity, and sustainability [6]. Medical management, aimed at reducing mortality and morbidity associated with dengue, requires considerable resource utilisation and poses a heavy economic cost and burden to the health systems of endemic countries [2]–[4]. The availability of a safe and effective vaccine against dengue would therefore have a substantial impact on the consequences of this disease.

Several dengue vaccine candidates are in clinical development [7], [8], and although many of these candidates are expected to progress in the future, currently only one has advanced to Phase III trials in support of licensure [9]. Case studies of other vaccine-preventable diseases, such as rotavirus and measles, have revealed unique challenges to introducing a vaccine in the developing world and highlighted the importance of early planning [10]. Early planning to support dengue vaccine introduction and evaluation is particularly important given that an effective dengue vaccine would have its greatest effect in developing countries, and only a limited effect in developed countries.

Dengue v2V (vaccine to vaccination) was established in 2009 to lay the groundwork for the accelerated introduction of effective dengue vaccines to endemic countries following regulatory approval. The aim of the initiative is to further our understanding of disease burden; raise awareness of the benefits of vaccination among regulatory agencies, national health authorities, and public health agencies; provide recommendations to national health authorities for vaccine introduction; and advocate for vaccine funding [11]. A 2010 v2V meeting produced a series of recommendations for dengue vaccine introduction in Southeast Asia [12].

With several dengue vaccine candidates on the horizon, the 1st Dengue v2V International Meeting, held in Puerto Rico in November 2011, examined challenges relating to dengue vaccine introduction and discussed the processes required to evaluate the outcomes of dengue vaccination programmes. Participants at the meeting included experts in dengue, vaccinology, and public health from 15 dengue-endemic countries. A series of plenary presentations provided experiences drawn from other vaccine-preventable diseases and pertinent to dengue vaccine, followed by structured discussions of the issues raised by these presentations. In addition, there was an interactive session on dengue surveillance systems in representative countries. The group made a number of recommendations for dengue vaccine introduction, which are presented in this report.

## Vaccine Introduction Case Studies

Experts from the rotavirus and measles fields presented case studies of the steps involved in establishing vaccination programmes and assessing their effectiveness. A key lesson from these studies was that early preparation is vital for successful vaccine introduction, particularly in developing countries where dengue is endemic. In particular, disease surveillance is essential to assess long-term safety and the impact of vaccination on disease, and needs to be well established at the prelicensure stage. Also, early preparations should determine cold chain storage capacity requirements, there should be early development of vaccinator training materials regarding the specific requirements of a new dengue vaccine, and the new vaccine must be included in systems that monitor vaccine coverage in the general population. Potential perceptual, political, or financial obstacles should be considered—for example, perceptual issues may include the perceived risk of adverse events or enhanced disease—and regional political leadership and social mobilisation, which have played a significant role in the introduction of several vaccines in Mexico [13], is crucial to overcoming these obstacles. Building an investment case that incorporates information regarding vaccine performance, cost-effectiveness, and disease burden, with input from public and nongovernmental organisations, was thought to be a critical step in the process of successfully introducing a dengue vaccine. Public events, such as immunisation days and health weeks, alongside media collaboration, have been found to be key strategies to engage the public, provide education on the benefits of vaccination, and address potential safety concerns.

The Pan American Health Organization (PAHO) has provided effective leadership and mechanisms such as a revolving fund to support the timely introduction of new vaccines among its member states. PAHO's ProVac initiative, which aims to strengthen countries' capacity for evidence-based decision making and provides a number of economic analysis tools, is a good example of a standardised approach to facilitating vaccine introductions [14], [15]. However, not all of these mechanisms exist in the World Health Organization (WHO) regional offices that cover Asia. It was the opinion of the group that WHO headquarters may need to facilitate dengue vaccine introduction through targeted activities in Asia where the majority of all dengue cases occur.

## Dengue Surveillance

Prevention and control programmes rely on surveillance to estimate disease burden, identify and possibly predict epidemics, monitor disease trends, and understand disease epidemiology. Furthermore, surveillance data allow interventions and prevention programmes to be evaluated and disease control objectives to be monitored. Most national dengue surveillance systems use passively reported case information, while some use sentinel surveillance systems located at designated institutions to collect data, which can then be applied to the national population. Data from dengue surveillance systems in several affected countries have shown wide variation in case classifications, surveillance methods, data analytic methods, and use of vector surveillance [16]. This highlights the significant limitations and the need for improved dengue surveillance systems in endemic countries.

### Recommendations for Surveillance Systems

Harmonisation of regional dengue case definitions and surveillance methodology would prepare countries for the dengue vaccine era and allow comparison of data between countries to assess vaccine impact. The ideal surveillance system would be nationwide, syndromic, have mechanisms to facilitate and stimulate high levels of reporting, include laboratory testing of at least a representative sample of cases (including infecting serotype), capture the final illness outcome (in terms of severity), and contain data on vaccination status. The system should be applied across all age groups, in outpatient and inpatient settings, and across both public and private sectors. One approach to national surveillance would be to have multiple sentinel sites that are representative of the country which measure laboratory-confirmed dengue incidence and severity to more accurately determine disease burden. Based on assessments of data quality and reliability (e.g., degree of underreporting), these data could then be extrapolated nationally. Long-term surveillance is important for assessing vaccination effectiveness, since data collected during interepidemic periods may provide greater predictive validity and, with a cyclical endemic disease such as dengue, it is necessary to know whether there has been suppression of the disease beyond historical lows. Following licensure, assessment of vaccine coverage will be crucial to determine the difference between failure to vaccinate, failure of vaccine strategy, or vaccine failure. Audit of the systems should be performed regularly to establish the integrity of the data and improve its reliability and quality.

Although a significant amount of local surveillance data are collected, currently there is limited global access to this data. Centralised access to disaggregated dengue surveillance data would aid decision making and public health action as local and global patterns could be identified more efficiently. Greater use of linked, electronic-based reporting systems would facilitate the flow of information from a local level to a global level. WHO involvement would facilitate the process and is therefore encouraged.

### Computational modelling

High-quality surveillance data are required to populate computational models, which are a useful tool for understanding DENV transmission, aiding the decision-making process, and exploring the impact of adding a dengue vaccine to national immunisation programmes. Modelling the supply chain of a vaccine can further help to plan and strategise vaccine introduction by showing the likely effects of changing logistical and environmental variables.

## Diagnostics

There is a need for specific, sensitive, and cost-effective diagnostic tests for dengue that can be used for clinical management and surveillance [17]. For clinical purposes, a diagnostic test should provide a rapid and dengue-specific diagnostic result in single acute-phase samples, while for epidemiological purposes the test should provide a reliable diagnosis of dengue and DENV serotype.

Dengue diagnostic testing is complicated by the appearance of potential analytes during the course of the disease [17]. DENV is present during the early phase of the illness and IgM anti-DENV becomes detectable later. Molecular diagnostics with nucleic acid amplification (e.g., polymerase chain reaction [PCR]-based tests) are currently considered the most sensitive techniques for diagnosis of dengue during the early acute-phase of the disease, followed by NS1 antigen detection. IgM anti-DENV becomes detectable as early as three days after the onset of illness in approximately 15% of patients, increasing to 56% by day five and 83% by day seven [18]. These tests are therefore important tools for patients presenting later in their illness. However, immune history may influence the performance of some tests, such as NS1 antigen and IgM anti-DENV, which are affected by whether the DENV infection is primary or secondary. IgG assays to detect anti-DENV in combination with other tests, such as neutralisation assays, are more suitable for studies of population immune status and are not useful for diagnosis of the acute illness [17]. While cross-reactivity with other flaviviruses is always a concern when DENV antigens are a component of the diagnostic test, misclassification rates have been substantially lowered in presently available tests [17].

A lack of laboratory capacity in some areas creates the need for rapid point-of-care diagnostic tests to provide clinically useful information or reference laboratory testing to obtain epidemiologic data. Capillary blood sampling with filter paper collection is a potential solution for sampling when resources are limited as filter paper can be transported at ambient temperature to an adequately equipped laboratory for diagnostic testing. This technique has an excellent overall performance, is inexpensive and simple to perform, and can be undertaken at ambient temperature [19], [20]. Dengue rapid diagnostic tests (IgM anti-DENV, NS1 antigen detection) also require less laboratory capacity. However, currently available tests do not perform as well as similar microtitre plate enzyme-linked immunosorbent assays (ELISAs), especially where dengue is endemic and there may be a high prevalence of secondary infection. While a lower test sensitivity may be adequate for epidemiologic surveillance, more work is necessary to develop rapid diagnostic tests for use in guiding clinical case management.

Studies are needed to establish a diagnostic testing algorithm combining viral and serological detection that achieves reliable dengue diagnosis for clinical as well as disease surveillance and vaccine evaluation purposes. Development of local laboratory capability will be a factor in the choice of diagnostic test format, and achieving optimal diagnostic accuracy will require capacity building in many regions. Effective dengue surveillance systems will require adoption of standardised diagnostic testing algorithms using tests that meet benchmarks for performance under good quality control with support from national and international reference laboratories.

## Establishing the Impact of Vaccination Programmes

The impact of a vaccination programme is determined by a number of factors, including vaccine efficacy and safety, vaccine coverage in the target population, and the effect of herd protection. A number of methods have been used to evaluate the impact of vaccines. In cluster-randomised trials, the phased introduction of a vaccine into a population allows for the unbiased assessment of its impact and is a powerful way to assess herd effects. Cohort studies are a valuable tool, but generally depend upon having population registries of disease occurrence and vaccination. Case-control studies provide a good way for assessing efficacy and controlling measurable confounding factors.

Extrapolation of data between countries is challenging, requiring consideration of international differences in surveillance, the epidemiology of the infection, and public health infrastructure. Furthermore, the effectiveness of a dengue vaccination programme is best determined in settings with high disease incidence and over extended periods of time because of population movements and cyclical variation in annual incidence, which may produce spurious results.

## Vaccination Safety

Assessing the safety profile of a vaccine requires significant surveillance and laboratory investment. Risks from dengue vaccines are believed to be mild and transient, though potentially large-scale vaccination could select for DENV vaccine escape variants or DENV variants with changed vector replication competence. Immune enhancement, another theoretical risk of vaccination, involves immune complex formation between heterotypic, nonneutralising antibodies from prior infection and the virus, leading to increased viral uptake and replication [21], [22]. A major challenge will be understanding how best to differentiate severe dengue that occurs following vaccination from naturally progressing severe dengue. To date, the development of a successful approach to address this problem has not been possible. There has been no safety signal with the lead vaccine candidate thus far [9], [23]. Due to the as yet undetermined risk window following vaccination, use of long-term surveillance, to track adverse events following immunisation (AEFIs) and link vaccination status to reported dengue cases, is more likely to inform safety assessments than hypothesis-driven studies.

Post-licensure, the safety of all aspects of immunisation needs to be monitored, including vaccine quality, transport, storage and handling, and administration. Active, rather than passive, surveillance of AEFIs is preferred. Data linking for AEFIs in the vaccinated population, such as that performed by the Brighton collaboration [24], should be considered.

## Phase IIb Data on Dengue Vaccination

Since the 1st Dengue v2V International Meeting, the results of a Phase IIb trial conducted in Thailand investigating the efficacy and safety of a leading candidate dengue vaccine have been published [23]. An excellent short-term safety profile was observed for the vaccine with efficacy demonstrated against DENV-1, -3, and -4 infection. However, there was no efficacy observed against DENV-2 infection (3.5% in the intention-to-treat analysis) in this setting despite the development of levels of vaccine-induced antibody previously thought to be protective, as determined by the plaque reduction neutralisation test (PRNT). The vaccine's protective effect against disease caused by the other three dengue serotypes (DENV-1 [61.2%], -3 [81.9%], and -4 [90.0%]) was achieved after at least one dose. However, the sample sizes were small and the trial was not designed to measure serotype-specific efficacy. Data from the ongoing Phase III multicentre, multicountry trial of this vaccine will determine the validity and importance of these preliminary findings and greatly inform future activities regarding dengue vaccine introduction. These initial findings further emphasise the need for DENV serotype-specific surveillance and the potential need for including molecular epidemiologic studies as part of vaccine-related dengue surveillance. Additional studies are also needed to better understand the immunological correlates of protection against disease and to model the potential use and effect of a partially effective vaccine.

## Conclusions

Dengue is a major global public health problem that should be amenable to control with introduction of a safe and effective vaccine. Early preparation is key to achieving these results and includes planning appropriate assessments of vaccine impact and safety. Improving and standardising diagnostics and surveillance systems are crucial steps to generating robust estimates of disease burden and ultimately determining vaccine impact, which will aid decision making regarding the use and introduction of a dengue vaccine. Further work is now needed to agree to best practices in dengue surveillance so that data can be compared between countries. As the vaccine will be introduced first in developing and middle-income countries, regulatory authorities will need effective systems in place for monitoring disease burden and AEFIs. Finally, a better understanding of the immunopathogenesis of dengue and factors influencing dengue disease severity will inform long-term assessment of vaccine impact and safety.
